# Bax mitochondrial relocation is linked to its phosphorylation and its interaction with Bcl-xL

**DOI:** 10.15698/mic2016.12.547

**Published:** 2016-12-05

**Authors:** David Garenne, Thibaud T. Renault, Stéphen Manon

**Affiliations:** 1 Institut de Biochimie et de Génétique Cellulaires, UMR5095, CNRS & Université de Bordeaux, CS61390, 146 Rue Léo Saignat, 33077 Bordeaux, France.; 2 Present address: INRA, UMR1332, 71 Avenue Edouard Bourlaud, 33882 Villenave d'Ornon, France.; 3 Present address: Department of Regulation in Infection Biology, Charitéplatz 1, 10117 Berlin, Germany.

**Keywords:** Bax, Bcl-xL, mitochondria, phosphorylation, cytochrome c release, apoptosis, yeast (S.cerevisiae)

## Abstract

The heterologous expression of Bax, and other Bcl-2 family members, in the yeast *Saccharomyces cerevisiae*, has proved to be a valuable reporter system to investigate the molecular mechanisms underlying their interaction with mitochondria. By combining the co-expression of Bax and Bcl-xL mutants with analyzes of their localization and interaction in mitochondria and post-mitochondrial supernatants, we showed that the ability of Bax and Bcl-xL to interact is dependent both on Bax phosphorylation - mimicked by a substitution S184D - and by Bax and Bcl-xL localization. This, and previous data, provide the molecular basis for a model of dynamic equilibrium for Bax localization and activation, regulated both by phosphorylation and Bcl-xL.

## INTRODUCTION

The pro-apoptotic protein Bax is at the core of the process of mitochondria-dependent apoptosis in mammals. Bax is a member of the Bcl-2 family, and contains 4 Bcl-2 homology domains (BH1 to BH4) that are involved in its interaction with other Bcl-2 family members, including the anti-apoptotic protein Bcl-xL. In non-apoptotic cells, Bax is generally expressed at a low level, and remains essentially cytosolic, or weakly associated to mitochondria 1(2,3 for reviews). Following an apoptotic stimulus, Bax is relocated to the mitochondria, and most specifically to the outer mitochondrial membrane. Bax can then be organized as dimers and oligomers that form a large-sized pore in this membrane. This pore favors the release of proteins together known as 'apoptogenic factors' that are released from the mitochondrial intermembrane space to the cytosol, and that confer their apoptotic characteristics to the cells. In addition to extended biochemical evidence, this model of a large sized pore formed with Bax molecules is also widely supported by electrophysiology 4,5,6, structural studies 7,8, biophysical approaches 9 and imaging data 10,11.

As a central event in the apoptotic process, the translocation of Bax from the cytosol to the mitochondria has been the focus of a large number of studies, but is still not completely resolved. When located in the cytosol, Bax is a globular and mostly hydrophilic protein 12. Bax can be activated through interacting with the BH3-domain of BH3-only proteins, such as tBid, Bim or Puma, that promotes major conformational changes to the protein 13. Structural studies suggested that the interaction between Bax and a BH3-domain induced the formation of a head-to-tail dimer 7, that is able to lay flat on the mitochondrial membrane 8. The association of several dimers is thought to form the oligomer that constitutes the pore 9. This hypothesis has been supported by microscopy experiments showing the formation of a large-sized pore both in membranes and in mitochondria *in situ* 10,11. However, this widely accepted model still contains a number of gray areas, including the role of the very hydrophobic C-terminal helix α9, that was absent from the structural data of the Bax dimer 7, and of which the actual role in Bax interaction with mitochondria remains unclear: indeed, its absence does not prevent the mitochondrial localization of Bax, nor Bax-induced outer membrane permeabilization 14,15.

One intriguing issue is the role of the serine residue in position 184 (S184). It is one of the few polar residues in this otherwise hydrophobic α-helix. The deletion of the S184 (ΔS184) converts α9 into a bona fide membrane anchor, that is able to drive the constitutive mitochondrial localization of Bax 16. Furthermore, the ΔS184 mutation prevented the regulation of mitochondrial Bax translocation by components of the TOM complex 17. It has been established that S184 can be phosphorylated by different kinases, such as AKT 18,19 and PKCζ 20. This phosphorylation was shown to impair the mitochondrial relocation of Bax during apoptosis, that is consistent with the pro-survival function of AKT, including in cancer cells 21. However, AKT has multiple cellular targets, and it is therefore difficult to identify precisely the actual role of Bax phosphorylation in the survival effects induced by AKT activation.

In recent experiments, we have co-expressed human Bax and AKT in yeast. Rather unexpectedly, we found that AKT increased both cellular and mitochondrial Bax content, and consequently increased the capacity of Bax to promote the release of cytochrome *c* 22. Also, substituted Bax mutants where S184 was replaced by non phosphorylatable Ala or Val residues, or by a phospho-mimetic Asp residue have been tested for their ability to interact with mitochondria 22,23. Like wild-type Bax, the phosphomimetic mutant S184D had the same weak mitochondrial localization that had already been observed in mammalian cells 18. However, this weak mitochondrial localization was paradoxically associated to a high capacity to release cytochrome *c*, suggesting that the mutation converted Bax into its active conformation 22,23. Conversely, the non-phosphorylatable mutants S184A and S184V had a much higher mitochondrial localization. Because of this, they induced a stronger release of cytochrome *c* than wild-type Bax. However, in spite of their much higher mitochondrial localization, they remained less active than the mutant S184D, suggesting that they adopted a poorly active conformation 22,23.

Bax translocation to mitochondria also depends on other proteins, such as the anti-apoptotic protein Bcl-xL. Under non-apoptotic conditions, Bcl-xL-overexpression increases the mitochondrial localization of Bax in parental mouse prolymphocytic cells FL5.12 or in human colorectal cancer cells HCT-116 24. A similar observation was made in yeast, where the co-expression of Bax and Bcl-xL induced a greater mitochondrial localization of Bax than when it was expressed alone 24. This suggests that Bax translocation was related to intrinsic characteristics of the interaction between Bax and Bcl-xL, that were independent from the mammalian cellular context. Furthermore, when a truncated mutant Bcl-xLΔC was co-expressed with Bax, the stimulation of Bax mitochondrial localization was even greater than wilth full-length Bcl-xL, both in mammalian cells and in yeast 24. Indeed, while Bcl-xL is able to retrotranslocate Bax from the mitochondria to the cytosol 25, Bcl-xLΔC lacks this ability 24,26 and stimulates Bax activation 24. These experiments and others 27 converge to support a model of dynamic equilibrium of Bax localization, that would be controlled, namely, by the phosphorylation of Bax and the interaction with Bcl-xL.

To further refine the model of regulation of Bax localization, we used yeast to investigate the dual role of Bax S184 phosphorylation and interaction with Bcl-xL on Bax sub-cellular localization and activation. Bax mutants on S184 were co-expressed with different Bcl-xL mutants, and their localization, activity and ability to interact with each other in each compartment, mitochondrial and extra-mitochondrial.

## RESULTS AND DISCUSSION

We previously reported that the phosphomimetic mutation S184D rendered Bax less stable in yeast, due to increased sensitivity to proteases: indeed, it was present at a lower level than BaxWT, but was restored at a normal level following the addition of the vacuolar protease inhibitor PMSF 22. To eliminate possible differences caused by this different susceptibility to proteolysis, all the following experiments were done in a strain carrying a deletion of *PEP4*, that encodes the yeast homolog of mammalian Cathepsin D, that is the most abundant and active yeast protease.

Δ*pep4* yeast strains co-expressing mutants Bax-S184D or S184A, and different variants of Bcl-xL were generated. Mitochondria and post-mitochondrial supernatants were isolated, and the presence of Bax and Bcl-xL was probed, to evaluate the extent of Bax mitochondrial relocation.

**Figure 1:**
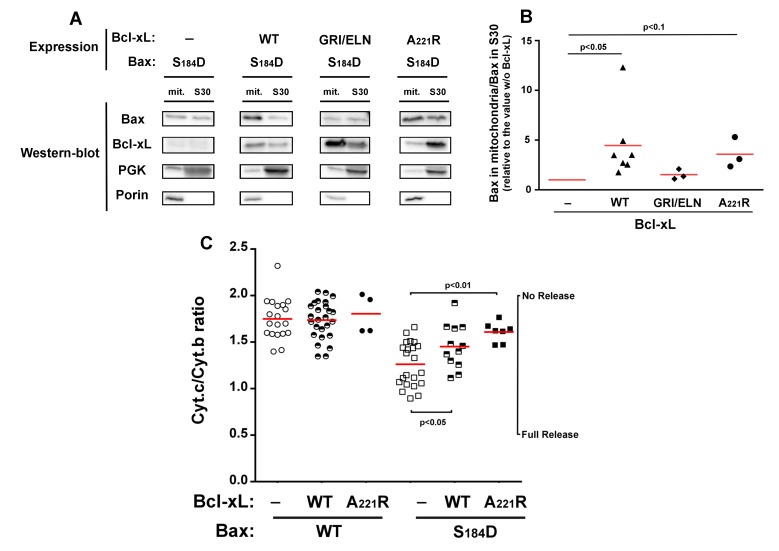
FIGURE 1: Effect of Bcl-xL on the localization and function of a Bax phosphomimetic mutant. Mitochondria (mit.) and post-mitochondrial supernatant (S30) were isolated from strains co-expressing Bax-S184D and wild-type Bcl-xL (WT), Bax interaction-deficient mutant carrying a triple mutation G138E/R139L/I140N (GRI/ELN), or a cytosolic mutant carrying a single mutation A221R (see Fig. 2). **(A)** Western-blot analysis of Bax and Bcl-xL localization in both fractions. **(B) **Quantification of the ratio between mitochondrial and non-mitochondrial Bax. All the experiments were done in parallel to a control without Bcl-xL, for which the ratio was adjusted to 1, as a reference. **(C)** Mitochondrial cytochrome *c* content, measured as the ratio cytochrome *c*/cytochrome b (unreleased cytochrome b serves as an internal control to the experiment). Each point represents an individual experiment. Data with wild-type Bax are given for a matter of comparison, and had been published previously 22,24,34, except for the results with wild-type Bax and Bcl-xL-A221R. Red bars indicates the averages. p values were calculated with an unpaired Student's test.

Like previously reported for BaxWT 24, the co-expression of full-length Bcl-xL stimulated the mitochondrial localization of Bax-S184D (Fig. 1A, B). As expected, a mutant of Bcl-xL carrying the triple substitution G138E/R139L/I140N impairing the interaction with Bax 24,28, did not increase mitochondrial Bax content (Fig. 1A, B). This unambiguously demonstrated that the physical interaction between Bax and Bcl-xL was required for the stimulating effect of Bcl-xL on Bax mitochondrial relocation.

**Figure 2:**
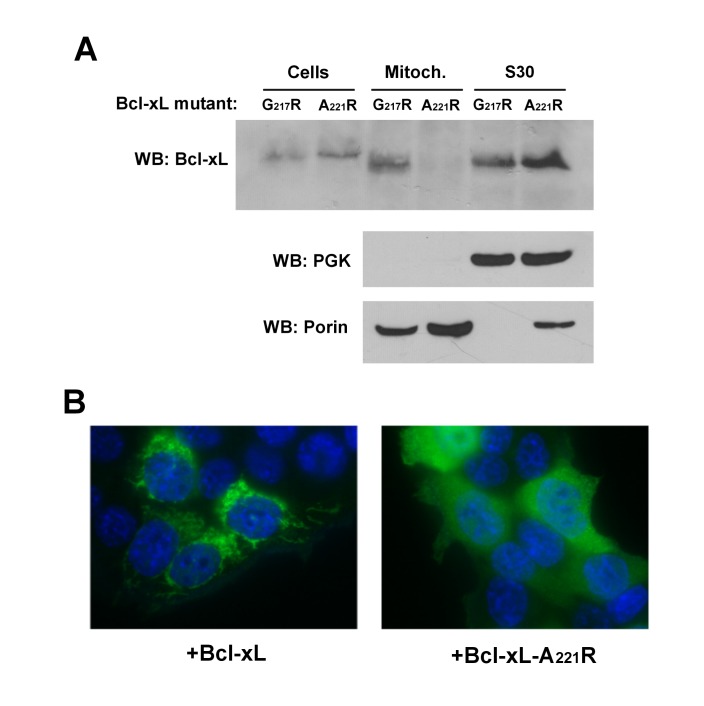
FIGURE 2: Characterization of a cytosolic mutant of Bcl-xL. **(A)** Localization of two mutants of Bcl-xL carrying single substitutions in the C-terminal α-helix, following their expression in yeast. The mutant A221R was excluded from the mitochondrial fraction, opposite to the mutant G217R. **(B) **Localization of wild-type and A221R mutant of Bcl-xL in HeLa cells. Cells were transfected with Bcl-xL-expressing plasmids and Bcl-xL was detected by immunofluorescence as described previously 35. Nuclei were counter-stained with DAPI. The mutant A221R displayed a diffuse cytosolic localization, opposite to the wild-type.

The C-terminal α-helix of Bcl-xL is crucial for the mitochondrial localization of Bcl-xL, and its deletion makes Bcl-xL lose its mitochondrial localization, both in yeast 29 and mammalian cells 30. However, the drastic deletion of the whole helix may have dramatic consequences on the overall structure of the protein, with rather unpredictable consequences. Indeed, we have observed that, when expressed alone in yeast, truncated Bcl-xLΔC had a weak but significant ability to release cytochrome *c* 24, that may be related to a non-selective interaction with membranes, caused by the exposure of the core of the protein that is normally masked by the C-terminal α-helix. To circumvent this possibility, we designed a mutant loosing the ability to reach mitochondria that was less drastically altered than the truncated protein. Residues at different positions in the C-terminal hydrophobic α-helix were substituted by large, positively charged residues (R or K), and the resulting mutants were tested for their capacity to be localized in the mitochondria. One mutant, carrying a substitution A221R, remained mostly cytosolic, both in yeast and human HeLa cells (Fig. 2).

When co-expressed with Bax-S184D, the mutant Bcl-xL-A221R remained cytosolic but stimulated the mitochondrial localization of Bax-S184D, although to a lower extent than wild-type Bcl-xL (Fig. 1A, B). The fact that, although interacting with Bax-S184D, Bcl-xL-A221R remained mostly cytosolic, indicated that a transient interaction between both proteins might be sufficient to promote the conformational change leading to Bax mitochondrial relocation. A similar behaviour had been previously suggested for Bcl-xLΔC, which increased the mitochondrial localization of Bax, although no stable interaction could be detected by co-immunoprecipitation 24.

Despite its weak mitochondrial localization, Bax-S184D has been shown to induce a large release of cytochrome *c* 22,23 (Fig. 1C). This shows that, even though a small amount of Bax-S184D can reach the mitochondrial membrane, it displays a very high capacity to permeabilize this membrane. On the other hand, it has been shown that AKT-dependent phosphorylation of Bax on S184 prevented apoptosis in human neutrophils 18. It can therefore be hypothesized that, under those conditions, additional factors were present, that impaired the capacity of phosphorylated Bax to reach and/or to permeabilize mitochondria.

Obvious candidates for this effect are anti-apoptotic proteins, such as Bcl-xL. Consequently, we investigated if the capacity of Bax-S184D to permeabilize mitochondria was or not sensitive to full-length Bcl-xL and to its cytosolic mutant Bcl-xL-A221R. As expected, cytochrome *c* release induced by Bax-S184D was partly inhibited by the co-expression of wild-type Bcl-xL (Fig. 1C), that is localized at the mitochondria (Fig. 1A, B). It could reasonably be expected that the largely cytosolic localization of Bcl-xL-A221R (Fig. 1A, B) would impair its ability to inhibit Bax. However, in striking contradiction with this prediction, and despite the fact that it stimulated the mitochondrial localization of Bax-S184D (Fig. 1A, B), we observed that Bcl-xL-A221R was able to inhibit Bax-S184D at least as efficiently as wild-type Bcl-xL (Fig. 1C).

**Figure 3:**
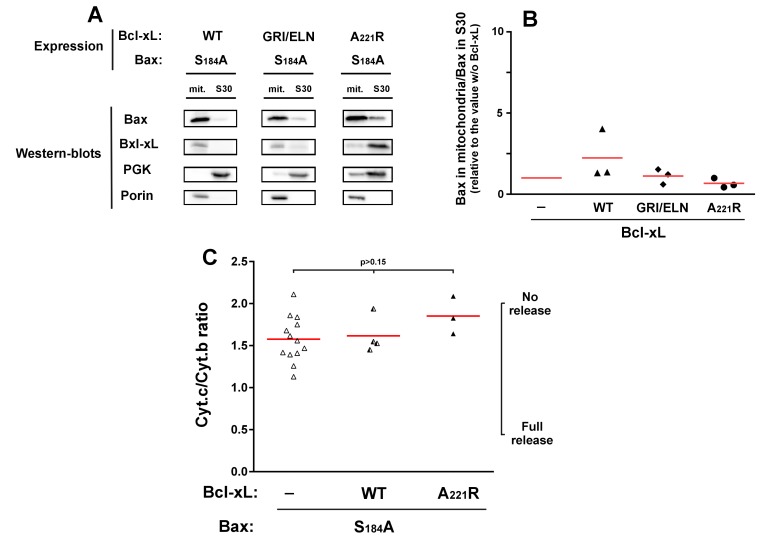
FIGURE 3: Effect of Bcl-xL on the localization and function of a non-phosphorylatable Bax mutant. Same conditions as in Figure 1, with Bax-S184A instead of Bax-S184D.

Although they are not perfect homologs, the yeast kinase Sch9p has similar functions as mammalian AKT 31. It is therefore possible that the expression of BaxWT in yeast would result in a partial phosphorylation of S184. We therefore expressed a non-phosphorylatable mutant Bax-S184A. This mutant had been found to have a constitutive mitochondrial localization both in neutrophils 18 and in yeast 22,23. We observed that this mitochondrial localization was insensitive to the presence of Bcl-xL, whichever mutant was used (wild-type, deficient for Bax-interaction, or cytosolic) (Fig. 3A, B). Despite this constitutive mitochondrial localization, and opposite to mutant Bax-S184D, Bax-S184A exhibited a weak capacity to release cytochrome *c* (Fig. 3C). Furthermore, this weak ability to release cytochrome* c* was not significantly inhibited by Bcl-xL (Fig. 3C), indicating that it was most likely related to the presence of a high amount of protein in the mitochondrial membrane, rather than to a bona fide specific activity.

**Figure 4:**
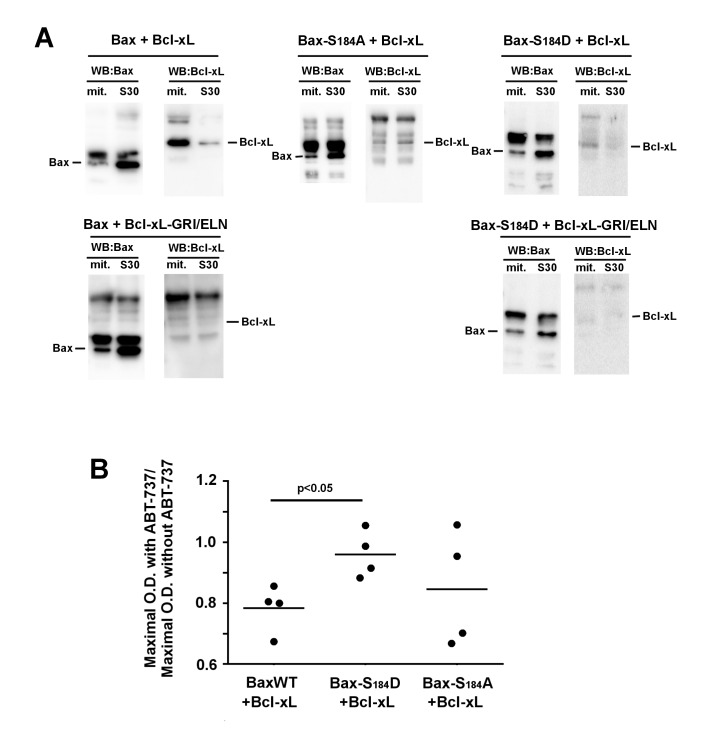
FIGURE 4: Interaction between Bax and Bcl-xL. **(A)** Immunoprecipitation experiments were done on mitochondria and S30 fractions from cells expressing indicated Bax mutants with or without Bcl-xL or Bcl-xL GRI/ELN, as a negative control. 2 mg proteins of each fraction were immunoprecipitated with an anti-Bax antibody (2D2, Sigma). Blots are representative of experiments that have been done 5 times for Bax/Bcl-xL, 3 times for Bax-S184A/Bcl-xL and Bax-S184D/Bcl-xL, and twice for negative controls with Bcl-xL-GRI/ELN. Inputs were omitted for clarity and can be seen in Fig. S1. **(Left) (Top)** The IP against Bax showed the interaction with Bcl-xL only in the mitochondrial fraction. **(Left) (Bottom)** **and** **(Right) (Bottom) **The absence of signal when BaxWT or Bax-S184D were co-expressed with Bcl-xL GRI/ELN evidenced the specificity of the signal. **(Middle)** Bax-S184A was co-expressed with wild-type Bcl-xL. The interaction was very weak, and similar in both fractions. **(Right)** Bax-S184D was co-expressed with wild-type Bcl-xL. Like for BaxWT, the interaction with Bcl-xL occurred mostly in the mitochondrial fraction. **(B)** Yeast cells co-expressing the three variants of Bax with Bcl-xL were grown in a small volume of SD-Lactate medium (2 mL) at the same O.D._600nm_ (0.2). 1% galactose was added to induce the expression of both proteins. 2 hours later, each culture was shared in half, and 20 µM ABT-737 (or an equivalent volume of DMSO) was added. The O.D._600nm_ was measured after 24 hours and the ratios between the presence and the absence of ABT-737 were plotted. The ratios did not change significantly after 36 hours.

We next investigated if the differential inhibition of Bax-S184D and Bax-S184A by Bcl-xL reflected a different degree of interaction between cytosolic and mitochondrial Bax. First, BaxWT and Bcl-xL were co-expressed, Bax was immunoprecipitated from the mitochondrial and post-mitochondrial fractions, and the presence of Bcl-xL was measured in the immunoprecipitate (Fig. 4A). We observed that the low amount of Bax present in mitochondria could immunoprecipitate a large amount of Bcl-xL. Conversely, the large amount of Bax present in the post-mitochondrial supernatant could only precipitate traces of Bcl-xL. As a negative control, no co-immunoprecipitation was observed between BaxWT and the interaction-deficient mutant Bcl-xL G138E/R139L/I140N.

The same experiment was done with the mutant Bax-S184A and Bcl-xL. In line with the fact that Bcl-xL did not inhibit the moderate cytochrome *c *release induced by Bax-S184A, no significant interaction could be detected between Bax-S184A and Bcl-xL in mitochondria, even though both proteins are abundantly present in this compartment.

The same experiment was done with the mutant Bax-S184D and Bcl-xL. Some weak interaction was found in mitochondria (compare with the total absence of signal in the control experiment with the mutant Bcl-xL G138E/R139L/I140N), while, in spite of the high content of both proteins, no interaction could be depicted in the post-mitochondrial supernatant.

In a previous paper, we used the BH3-mimetic molecule ABT-737 to reveal the priming of Bax by Bcl-xL in yeast 24, in an assay that was based on a similar experiment done on pure recombinant proteins 32. We had found that, in the presence of Bcl-xL, ABT-737 activated BaxWT while it was without effect on BaxWT expressed alone, which was interpreted as the fact that the interaction of Bax with Bcl-xL favored the active conformation of Bax. We replicated the experiment with BaxWT/Bcl-xL, but not with Bax-S184D/Bcl-xL (Fig. 4B), that was in line with the hypothesis that Bax-S184D is already under an active conformation. However, we observed an ambiguous result with Bax-S184A/Bcl-xL, suggesting that this mutant might still be converted to an active conformation, although less easily than BaxWT, that is in line with the observation above that it interacted loosely with Bcl-xL (Fig. 4A).

**Figure 5:**
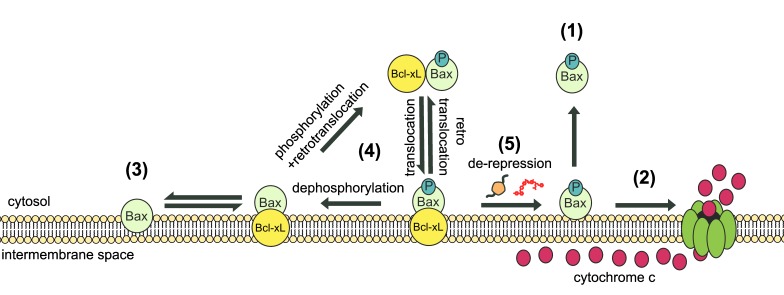
FIGURE 5: Model of dynamic equilibrium for Bax localization and activation. The model is based on data from the present paper, and from previous papers on the yeast model 20,22, and on mammalian cells 24,26,27. **(1)** When Bax is phosphorylated on S184, it is spontaneously mostly located in the cytosol. However, the small fraction that remains in the mitochondrial membrane is able to oligomerize to form a pore that promotes the release of cytochrome *c* **(2)**. **(3)** When Bax is not phosphorylated, it is spontaneously mostly located in the mitochondrial membrane, but is unable to oligomerize to form the pore. **(4) **In the presence of Bcl-xL, phosphorylated Bax and Bcl-xL are conveyed together to the membrane where the high stability of the interaction prevents the activation of Bax. Although the process is reversible through retrotranslocation, the system is favored towards Bax mitochondrial localization through the possible dephosphorylation of Bax. **(5)** Conversely, in the presence of derepressors of the interaction between Bax and Bcl-xL (BH3-only proteins, such as Bim, or BH3-mimetic molecules, such as ABT-737), Bax is able to form a pore with great efficiency because it is already present in great amount in the membrane (see the discussion in 24).

Taken together, data reported herein suggest that the phosphorylation status of Bax S184 residue is crucial to determine its ability to relocate at the outer mitochondrial membrane, to permeabilize this membrane, and to be inhibited by Bcl-xL. A tricky aspect of Bax properties is that, when considered separately, they may have contradictory outcomes on the final activity of Bax, i.e. on its ability to promote the release of cytochrome *c* (and possibly of other factors) and to trigger apoptosis. Indeed, mimicking the phosphorylation of S184, that may occur following the activation of the survival (i.e. anti-apoptotic) protein kinase AKT, has distinct consequences, depending on the presence of Bcl-xL. In the absence of Bcl-xL, Bax-S184D had a strong activity that is not abolished by its weak mitochondrial localization. This resulted in a high capacity to permeabilize mitochondria. However, when Bcl-xL is also expressed, a condition that most likely reflects the situation in tumoral cells, Bax-S184D is both relocated to the mitochondria and bound and inhibited by Bcl-xL. We have previously demonstrated that, in mammalian cells, this situation put the cells on the edge of entering apoptosis, simply by breaking the interaction between Bax and Bcl-xL with, for example, a BH3-mimetic drug, that mimics the action of a BH3-only protein, such as Bim, on prolymphocytes 24. Alternatively, if the interaction between Bax and Bcl-xL is maintained, due to the absence/inactivation of BH3-only proteins, Bax can be retrotranslocated to the cytosol (Fig. 5).

Another aspect to consider is the higher turnover of Bax-S184D (compared to BaxWT) that reflects a greater protease sensitivity 22. In the present study, we have reduced the influence of this parameter by working in a Δ*pep4* strain, allowing us to detect the greater intrinsic activity of this mutant, that might not have been possible in situations where this mutant was quickly degraded. Furthermore, Bcl-xL protects Bax-S184D against proteases, by masking proteolytic sites 22 and/or driving Bax to the mitochondrial outer membrane 24, a process that may also happen in mammalian cells 24 and be extended to Bcl-2 33. This might also explain why Bax phosphorylated on S184 is not easily detectable.

The introduction of a negative charge in an overall hydrophobic α-helix is expected to impair the membrane insertion of this helix. It is therefore somewhat counter-intuitive that Bax-S184D is able to release cytochrome *c *(Fig. 1). However, the recent models of Bax pore do not any give indication about the localization of α9 (because it is absent from the resolved structure) and it might be speculated that it could be localized on the walls of the pore, with the negative charge facing the hole, thus facilitating the passage of positively charged cytochrome *c*. As a matter of fact, we have reported that a mutant carrying a mutation T174D, located on the same side of the helix as S184, had a cytochrome *c* release activity when expressed in yeast 34.

Mimicking the total absence of phosphorylation on S184, has completely different consequences: Bax-S184A has a constitutive mitochondrial localization, is poorly active, and both its localization and activity are insensitive to Bcl-xL. It should be noted, however, that Bax-S184A and Bax-S184D are not mirroring each other: both are more efficient than wild-type Bax to induce cytochrome *c* release, the former because of its higher mitochondrial content, the later because of its higher intrinsic activity. This may indicate that non-phosphorylated Bax can be located at the outer mitochondrial membrane, independently from the presence of Bcl-xL 22, but can only be fully activated if Bax is phosphorylated, so that the whole previous cycle can occur (Fig. 5). This intricate succession of events, combining Bax phosphorylation and dephosphorylation, mitochondrial relocation and retrotranslocation, and binding and release to Bcl-xL (Fig. 5), may ensure a finely tuned regulation of cell death.

## Materials and Methods

The *Saccharomyces cerevisiae* strain W303-1A (*mat a; ade2-1; his3-11,15; leu2-3,112; trp1-1; ura3-1*) was carrying a deletion of the gene *PEP4*, of which the entire open reading frame was replaced by the gene *kanMX4*. This was done to prevent the degradation of the Bax-S184D mutant 22. This strain W303-1A Δ*pep4::kanMX4* was transformed or co-transformed with pYES3 and pYES2 plasmids that drove the expression of Bax and Bcl-xL mutants, respectively. For Bax, the cDNA encoding the complete human Bax (without additional tag) was modified to fit the yeast codon bias. Bax and Bcl-xL were cloned downstream the GAL1/10 promoter. Mutations were introduced by the Quickchange method, and the complete sequence of Bax and Bcl-xL was checked for the absence of unwanted mutations.

Yeast cells were grown aerobically in a minimal medium (0.17% Yeast Nitrogen Base, 0.5% ammonium sulfate, 0.1% potassium phosphate, 0.2% Drop-Mix, 2% DL-Lactate, 0.01% auxotrophic requirements, pH 5.5) until early exponential growth phase (O.D._550nm_ = 0.5). 1% galactose was added to induce the expression of Bax and Bcl-xL for 14 hours. At this stage, the O.D. of yeast cultures were in the range 1 ~ 1.5. Mitochondria and post-mitochondrial supernatants (S30) were isolated from spheroplasts, like published previously 34. Most experiments have been done immediately on freshly isolated fractions, but some additional experiments have been done on mitochondrial suspension frozen as small beads in liquid nitrogen.

For measuring Bax and Bcl-xL content, 0.5 mg proteins from mitochondria and S30 have been precipitated with 0.3M trichloroacetic acid, washed twice with acetone, and solubilized in Laemmli buffer. 100 µg of proteins were separated by SDS-PAGE (12.5% acrylamide), blotted on PVDF, and revealed with antibodies against Bax, Bcl-xL, Porin and PGK (as mitochondrial and cytosolic markers, respectively).

For immunoprecipitation, 2 mg proteins from mitochondria and S30 fractions were solubilized for 30 minutes in 0.5 mL of 1 X IP50 buffer from Sigma, then incubated overnight with 2 µg anti-Bax antibody (2D2, Sigma), and 4 additional hours with protein 50 µL G-sepharose beads (Sigma). Beads were washed 4 times with 1 X IP50 buffer and twice with 0.1 X IP50 buffer, and incubated with 20 µL Laemmli buffer, before SDS-PAGE and Western-blotting.

Cytochromes content of mitochondria was measured by differential redox spectrophotometry, as described previously 24.

Antibodies used were as follows: rabbit polyclonal anti-human Bax N20 antibody (Santa-Cruz, 1/5,000e), mouse monoclonal anti-human Bcl-xL antibody (BD Transduction Laboratories, 1/5,000e), mouse monoclonal anti-yeast porin (Novex, 1/40,000e), mouse monoclonal anti-yeast PGK (Novex, 1/10,000e), HRP-coupled anti-rabbit or mouse IgG (Jackson Laboratories, 1/10,000e).

Western-blot and immunoprecipitation experiments were done a minimum of 5 times, on mitochondria and S30 fractions done simultaneously on strains relevant for direct comparison (for example, 3 strains co-expressing one Bax mutant with 3 mutants of Bcl-xL, or 3 strains co-expressing 3 Bax mutants with the same mutant of Bcl-xL). ABT-737 was from Abbott Labs and was dissolved in cell culture-grade DMSO.

## SUPPLEMENTAL MATERIAL

Click here for supplemental data file.

All supplemental data for this article are also available online at http://microbialcell.com/researcharticles/bax-mitochondrial-relocation-is-linked-to-its-phosphorylation-and-its-interaction-with-bcl-xl/.
