# Supplementary material for: Bax mitochondrial relocation is linked to its phosphorylation and its interaction with Bcl-xL
Source: Microb Cell. 2016 Dec 5;3(12):597–605. doi: 10.15698/mic2016.12.547 (PMC5348979; doi:10.15698/mic2016.12.547)
Supplement: Supplementary file 1 [file mic-03-597-s01.pdf]

Figure S1: Interaction between Bax and Bcl-xL

Experiments were done on mitochondria and S30 fractions from cells expressing Bax mutants with or without Bcl-xL. 2mg proteins of each fraction were immunoprecipitated with an anti-Bax antibody (2D2, Sigma). Typically, 100µg (5%) of the initial fractions were loaded in the 'extract' wells, and 25% of the immunoprecipitated fraction were loaded in the 'IP:Bax' wells. Each experiment has been done at least three times with similar results.

(A) (Top) Wild-type Bax was co-expressed with wild-type Bcl-xL. The IP against Bax showed the interaction with Bcl-xL only in the mitochondrial fraction (IP:Bax; WB: Bcl-xL). (bottom) The absence of signal when BaxWT was co-expressed with Bcl-xL GRI/ELN evidenced the specificity of the signal.

(B) Bax-S184A was co-expressed with wild-type Bcl-xL. The interaction was very weak, and similar in both fractions.

(C) Bax-S184D was co-expressed with wild-type Bcl-xL. Like for BaxWT, the interaction with Bcl-xL occurred mostly in the mitochondrial fraction.

(A)

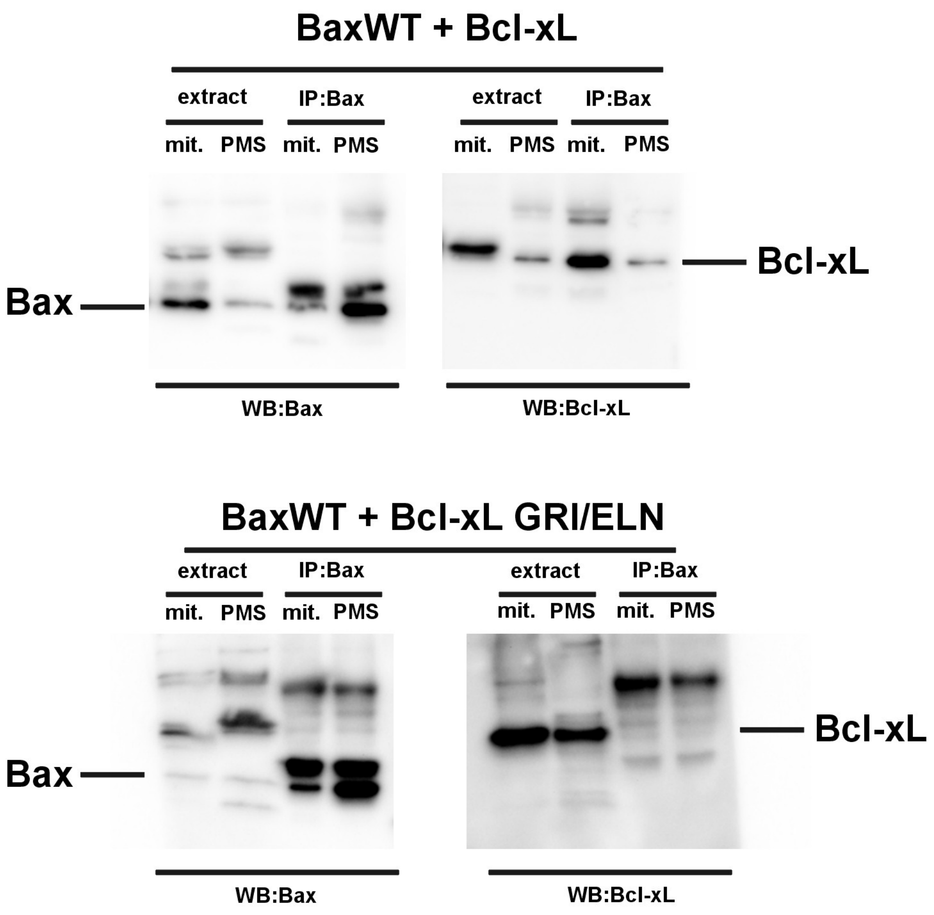

(B)

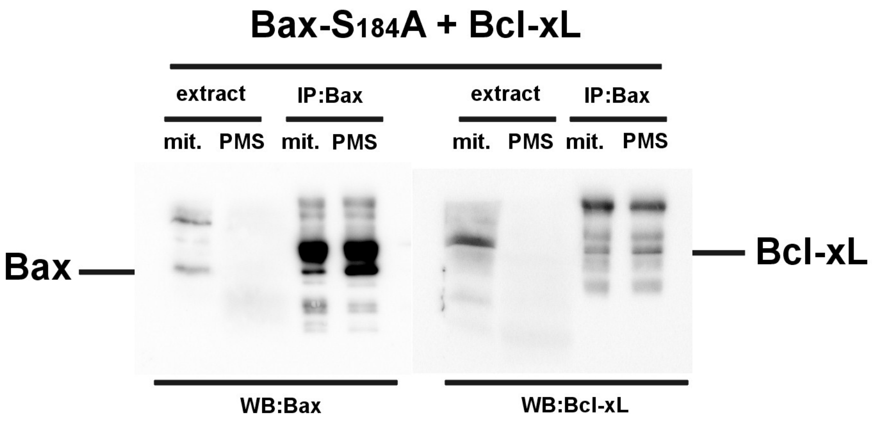
Bax-S184A + Bcl-xL
extract
IP:Bax
mit.
PMS
mit.
PMS
extract
IP:Bax
mit.
PMS
mit.
PMS
Bax
Bcl-xL
WB:Bax
WB:Bcl-xL

(C)

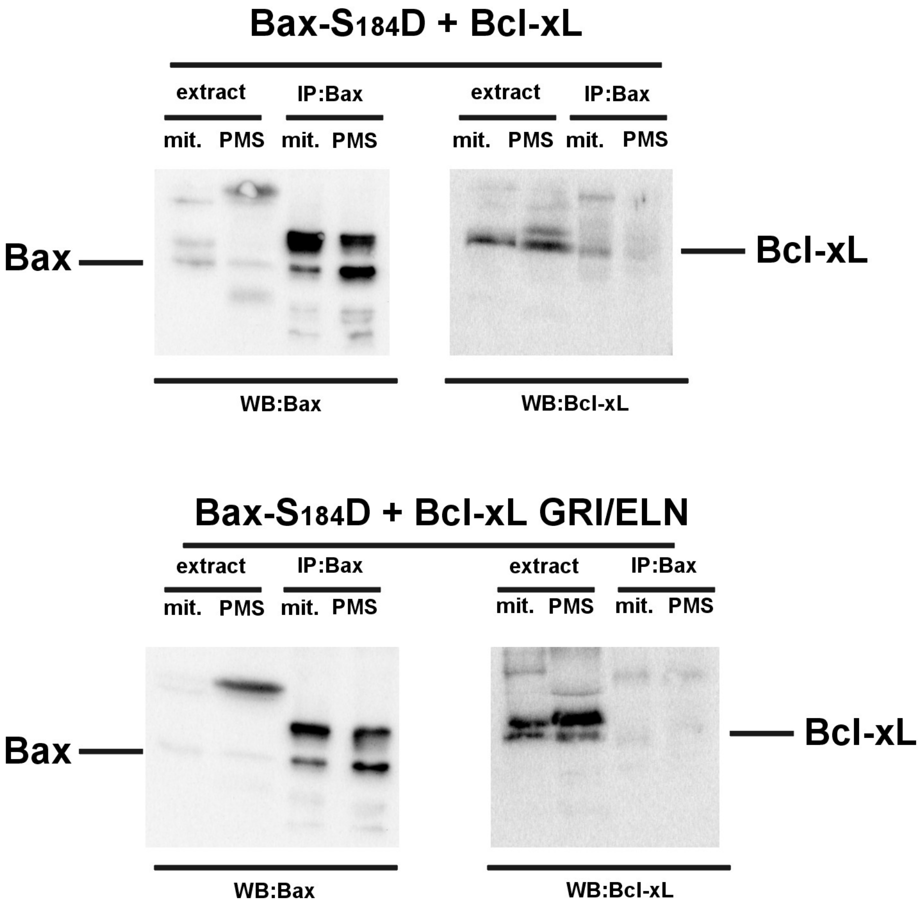
Bax-S184D + Bcl-xL
extract
IP:Bax
mit.
PMS
mit.
PMS
extract
IP:Bax
mit.
PMS
mit.
PMS
Bax
Bcl-xL
WB:Bax
WB:Bcl-xL
Bax-S184D + Bcl-xL GRI/ELN
extract
IP:Bax
mit.
PMS
mit.
PMS
extract
IP:Bax
mit.
PMS
mit.
PMS
Bax
Bcl-xL
WB:Bax
WB:Bcl-xL
